# Light-Chain Multiple Myeloma: A Diagnostic Challenge

**DOI:** 10.7759/cureus.19131

**Published:** 2021-10-29

**Authors:** Cristina Silva, Ana Costa, David Paiva, Sara Freitas, Glória Alves, Jorge Cotter

**Affiliations:** 1 Internal Medicine, Hospital Senhora da Oliveira, Guimarães, PRT

**Keywords:** lytic bone lesion, renal insufficiency, anemia, free light chains, multiple myeloma

## Abstract

Light-chain multiple myeloma (LCMM) is a less frequent type of multiple myeloma (MM), with a more aggressive course and poorer prognosis. It is characterized by the inability of the malignant plasma cells to produce heavy chains, resulting in the exclusive production of light chains. Therefore, no M-spike is visible in serum protein electrophoresis. We described the case of a 67-year-old female who presents to the emergency department with anemia, severe renal insufficiency, and multiple lytic bone lesions. After three days, the diagnosis of kappa light chain multiple myeloma was made in a patient with elevated serum and urinary kappa light chains and a bone marrow aspirate with 21.7% of atypical plasma cells. The rapid diagnosis allowed prompt referral to a specialized multiple myeloma center and early initiation of treatment.

## Introduction

Multiple myeloma (MM) is the second most common hematologic malignancy and represents 1% of all cancers. The prevalence is higher in men and African-Americans. The median age at diagnosis is about 65 years [1-4]. Clinical manifestations include anemia, renal failure, lytic bone lesions, hypercalcemia, susceptibility to infection and, occasionally, clotting abnormalities, neurologic symptoms, and manifestations of hyperviscosity [1-2,5].

MM is a malignant lymphoproliferative B-cell disorder characterized by an abnormal clonal proliferation of plasma cells [1,2]. Normal plasma cells produce immunoglobulins formed by a heavy and a light chain. Malignant clonal plasma cells secrete an excess of either intact immunoglobulins or free light chains of a single type, called monoclonal proteins (M-protein) [6]. The most common type of M-protein is immunoglobulin (Ig)G followed by IgA and light chain only [2,4]. The diagnosis of MM is established when at least 10% clonal plasma cells are present in the bone marrow or a biopsy-proven plasmacytoma is found, and at least one myeloma-defining event is present. Myeloma-defining events include evidence of end-organ damage (hypercalcemia, renal insufficiency, anemia, or bone lesions) or biomarkers of malignancy (≥ 60% clonal bone marrow plasma cells, involved/uninvolved serum free light chain ratio ≥ 100 or > 1 focal lesion on MRI studies of ≥5 mm in size) [7].

In light-chain multiple myeloma (LCMM), which accounts for 15% of MM cases, clonal plasma cells are unable to produce heavy chains, resulting in the exclusive production of light chains [2]. LCMM has a more aggressive course and poorer prognosis when compared to other types of MM. Renal failure, bone disease, and systemic light-chain AL amyloidosis appear to be more frequent [2,4]. Regarding treatment, bortezomib regimens have shown superior efficacy in LCMM when compared to nonbortezomib regimens [2,4].

## Case presentation

A 67-year-old female went to the emergency department for back pain that started a week before, with progressive worsening and inability to do the usual housework, despite tramadol/paracetamol 75/650 mg tid. She also mentioned decreased urinary output, anorexia, and low water ingestion starting at the same time. She denied fever, nausea, vomit, dysuria, urinary frequency or urgency, or respiratory symptoms. Her past medical history included hypertension, dyslipidemia, depression, peripheral vertigo, and esophagitis. At admission, she was slightly dehydrated, pale, and had pain on the palpation of the lumbar spine. She was hemodynamically stable and afebrile, without any other remarkable feature on physical examination. Laboratory investigations in the emergency department are shown in Tables 1 and 2. It was documented anemia of chronic disease, leukocytosis with neutrophilia, high C-reactive protein, high erythrocyte sedimentation rate, hypoalbuminemia, renal insufficiency, high anion-gap metabolic acidosis, ionized calcium level in the upper normal limit, hyperphosphatemia, and elevated lactate dehydrogenase levels. Urinalysis showed leukocyturia. The chest radiograph had no abnormal findings. Abdominal computed tomography (CT) showed normal kidneys, no ureterohydronephrosis, but lytic lesions in the iliac bones and lumbar vertebrae were visible. At this point, she was admitted to the Internal Medicine Department for additional study and treatment.

**Table 1:** Main laboratory test results in the emergency department.

Test result		Reference value
Hemoglobin (g/dL)	9.2	12.0–16.0
Mean corpuscular volume (fL)	85.4	83–103
Mean corpuscular hemoglobin (pg)	29.3	28–34
Mean corpuscular hemoglobin concentration (g/dL)	34.3	32–36
White blood cells	12.6 × 10^3^/μL	4.8–10.8 × 10^3^/μL
Neutrophils	10.3 × 10^3^/μL	1.8–7.7 × 10^3^/μL
Eosinophils	0.1 × 10^3^/μL	0.00–0.49 × 10^3^/μL
Basophils	0.0 × 10^3^/μL	0.0–0.1 × 10^3^/μL
Lymphocytes	1.0 × 10^3^/μL	1.0–4.8 × 10^3^/μL
Monocytes	1.1 × 10^3^/μL	0.12–0.80 × 10^3^/μL
Platelets	203 × 10^3^/μL	150–350 × 10^3^/μL
Reticulocyte count	0.0286 × 10^6^/μL	
Erythrocyte sedimentation rate (mm/h)	64	0–35
C-reactive protein (mg/L)	270	<3.0
Urea (mg/dL)	170	15–39
Creatinine (mg/dL)	7.20	0.57–1.11
Sodium (mEq/L)	135	135–146
Potassium (mEq/L)	5.79	3.5–5.1
Phosphorus (mg/dL)	7.0	2.5–4.9
Total bilirubin (mg/dL)	0.32	0.2–1.0
Lactate dehydrogenase (UI/L)	290	84–246
Aspartate aminotransferase (UI/L)	20	15–37
Alanine aminotransferase (UI/L)	37	30–65
Albumin (g/dL)	2.7	3.4–5.0
Iron studies		
Serum iron (g/dL)	9	50–170
Serum transferrin (mg/dL)	204	200–360
Total iron binding capacity (g/dL)	241	250–450
Serum ferritin (ng/mL)	352	8–252
Calculated transferrin saturation (%)	3.7	
Free T4 (ng/dL)	1.36	0.76–1.46
TSH (UI/mL)	0.596	0.358–3.740
Acid folic (ng/mL)	15.3	3.1–17.5
Vitamin B12 (ρg/mL)	443	211–911

**Table 2:** Arterial blood gas analysis in the emergency department.

Arterial blood gas analysis		Reference value
pH	7.349	7.350–7.450
pCO^2^ (mmHg)	30.4	35.0–45.0
pO^2^ (mmHg)	98.1	80.0–100.0
$\mathrm{HCO}^{_{3}^{-}}$ (mmol/L)	16.4	22.0–31.0
K^+^ (mmol/L)	5.01	3.50–5.10
Na^+^ (mmol/L)	133.6	135.0–145.0
Cl− (mmol/L)	101.0	87.0–106.0
Ca^2+^ (mmol/L)	1.29	1.15–1.35

**Figure 1:**
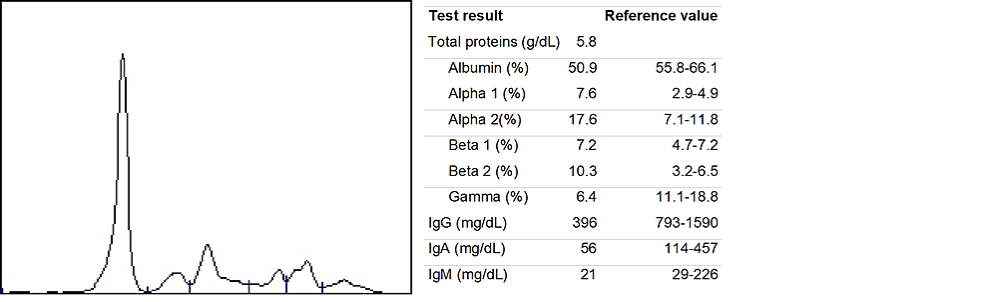
Serum protein electrophoresis showing a flattening of the gamma zone in a patient with LCMM and immunoparesis.

**Figure 2:**
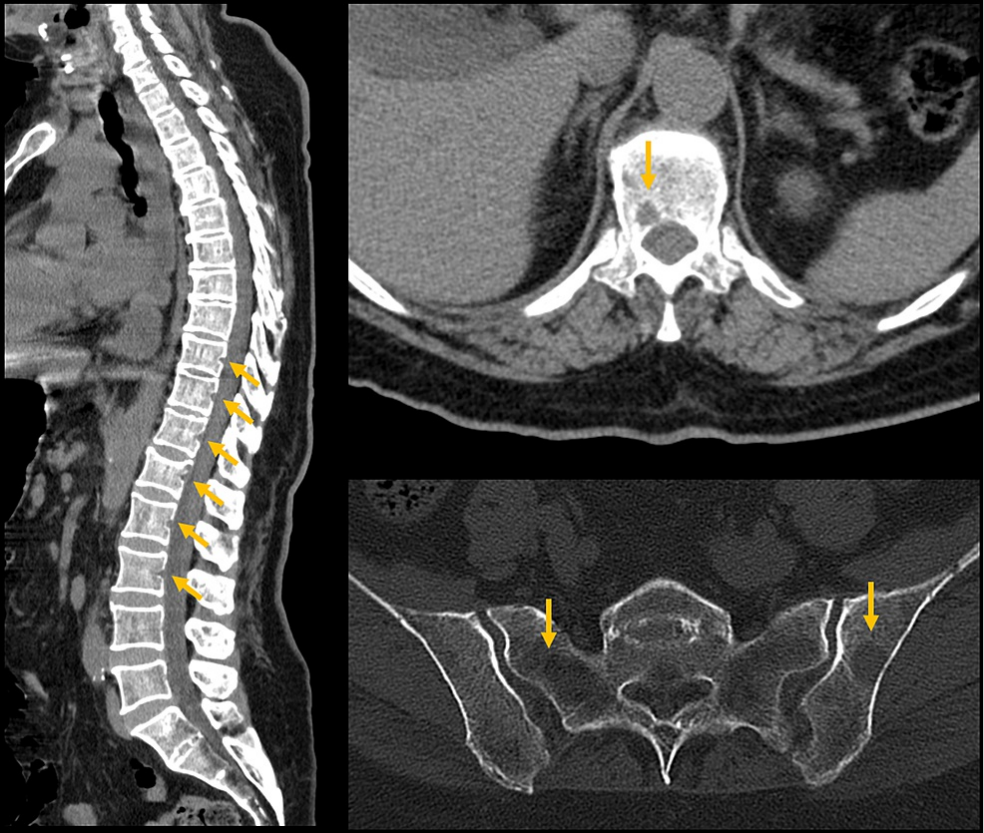
Lumbar CT showing multiple lytic lesions in vertebral bodies, sacrum, and iliac bones (yellow arrows).

Facing a patient with renal insufficiency, anemia, and lytic bone lesions, our main diagnostic hypotheses were multiple myeloma and solid metastatic malignant neoplasm (breast, lung, thyroid). A battery of examinations was set in motion on the first day of hospital admission (D1) to answer these clinical questions. Serum protein electrophoresis revealed a flattening of the gamma zone (Figure 1). Quantification of immunoglobulins showed low levels of IgG, IgA, and IgM. Serum immunoelectrophoresis had no abnormalities. Given the high clinical suspicion of MM, even with an absent M-spike on serum protein electrophoresis, and because quantification of serum and urinary-free light chains would take 72 hours to be available, we decided to perform bone marrow aspiration on D1. CT spine was ordered to characterize bone lesions and showed multiple small scattered lytic lesions in the vertebrae, sacrum, and iliac bones (Figure 2). A slight depression of the upper platform of D12 with solutions of bone continuity suggested a recent fracture.

The patient received fluid therapy and transdermal buprenorphine 35 μg/h patch every-three-day for pain control. According to orthopedics, vertebra fracture was stable, with the indication for Jewett brace and no need for surgical treatment. On D4, the patient was lethargic and had asterixis. Urea and creatinine levels were rising (urea 216 mg/dL, creatinine 7.90 mg/dL). No fever or focal neurological signs were present. Head CT had no abnormalities. This altered level of consciousness was interpreted as uremic encephalopathy. Bone marrow aspirate report was available on that day and showed 21.7% of plasma cells with atypical morphology. Serum kappa light chains levels were elevated (408 mg/dL, N 170-370), and the kappa/lambda ratio was 8.95 (N 1.35-2.70). Urinary kappa light chains were 40 mg/dL compared with lambda light chains of 0.6 mg/dL.

Diagnosis of kappa light chain MM was established, and the patient was transferred on the same day to a Hematology Referral Center. She was on stage III of the International Staging System for MM based on the value of β2 microglobulin (22.9 mg/L). Three days after being transferred, she started treatment with bortezomib-dexamethasone. She had a favorable response, no need for dialysis, and was discharged after three weeks with a creatinine level of 1.6 mg/dL. Nevertheless, in the following months, the patient showed progression of the disease despite three lines of chemotherapy and died 29 months after the diagnosis.

## Discussion

LCMM represents only 15% of MM cases, and the literature data are scarce. Clinical characteristics are similar to other types of MM. The most common signs and symptoms at presentation are bone pain and renal failure. Lytic bone lesions, hypercalcemia, anemia, pleural effusion, and extramedullary disease can be found over the course of the disease [2,4].

The most distinctive feature of LCMM is the absence of complete clonal immunoglobulins secretion by the malignant plasma cells [2]. This is important because serum electrophoresis has no M-spike, which can lead to wrongly rule-out the diagnosis. Hence, when there is clinical suspicion of MM, assessment of serum and urinary free light chains should be part of the initial workup.

Renal involvement is more common in LCMM patients compared to other MM types [2,4]. Renal impairment at diagnosis is associated with inferior survival, significant morbidity, and an increased early death rate [8-10]. Supportive measures and the immediate institution of antimyeloma therapy are the mainstays of therapy for patients with MM and renal insufficiency [11]. Bortezomib-based regimens are the cornerstone of the management of myeloma-related renal insufficiency [8-11]. In LCMM, they also showed superior efficacy, with median survival times of 23 months in patients treated with bortezomib and 12 months in patients without [2,4].

We described the case of a 67-year-old female with anemia, severe renal insufficiency and uremic encephalopathy, and multiple lytic bone lesions. Our main diagnostic hypothesis was MM. Serum protein electrophoresis had a flattening of the gamma zone, no M-spike was visible. Given the high clinical suspicion and the severity of renal insufficiency, we did not want to wait for the result of serum and urinary light chain analysis, so we performed bone marrow aspiration on the first day. After three days, the results were available and confirmed the diagnosis of kappa LCMM. This allowed prompt referral to a specialized MM treatment center and early initiation of treatment.

## Conclusions

LCMM is an infrequent type of MM characterized by the absence of complete clonal immunoglobulins secretion by the malignant plasma cells. There are excessive serum clonal light chains, but serum protein electrophoresis has no M-spike. The clinician should be aware of this entity when evaluating patients with signs and symptoms suggestive of MM, such as anemia, renal failure, bone pain, and lytic lesions. LCMM has a worse prognosis than other more common MM types, so a rapid diagnosis and early treatment are vital for improving patient outcomes.
